# 

**Published:** 1916-09

**Authors:** 


### Contents

Event and Comment .... 401
Pyorrhea Alveolaris .... 404
Value of Oral Hygiene Lectures .... 417
Tuberculosis of the Mouth .... 423
Synthetic Porcelain .... 426
Treatment of Pyorrhea Alveolaris .... 429
Permeability of Normal Mucous Membrane .... 433
Dental Hygienist and Preventive Dentistry .... 434
Tristate Meetings at Kansas City .... 437
Preventive Dentistry .... 438
Commercialism in Dentistry .... 440
Agreements to Surrender Practice .... 441
Indents .... 443
Bibliography .... 449

